# A Natural Bacterium-Produced Membrane-Bound Nanocarrier for Drug Combination Therapy

**DOI:** 10.3390/ma9110889

**Published:** 2016-11-02

**Authors:** Ruimin Long, Yuangang Liu, Qinglei Dai, Shibin Wang, Qiongjia Deng, Xia Zhou

**Affiliations:** 1 College of Chemical Engineering, Huaqiao University, Xiamen 361021, China; simplever@126.com (R.L.); m18968015279@163.com (Q.D.); sbwang@hqu.edu.cn (S.W.); dengqiongjia@126.com (Q.D.); josiechou1993@163.com (X.Z.); 2 Institute of Pharmaceutical Engineering, Huaqiao University, Xiamen 361021, China; 3 Fujian Provincial Key Laboratory of Biochemical Technology, Huaqiao University, Xiamen 361021, China

**Keywords:** magnetosomes, natural carrier, drug combination therapy, dual crosslinkers

## Abstract

To minimize the non-specific toxicity of drug combination during cancer therapy, we prepared a new system synthesized from bacteria to deliver the anticancer drugs cytosine arabinoside (Ara-C) and daunorubicin (DNR). In this study, we selected genipin (GP) and poly-l-glutamic acid (PLGA) as dual crosslinkers. Herewith, we demonstrated the preparation, characterization and in vitro antitumor effects of Ara-C and DNR loaded GP-PLGA-modified bacterial magnetosomes (BMs) (ADBMs-P). The results show that this new system is stable and exhibits optimal drug-loading properties. The average diameters of BMs and ADBMs-P were 42.0 ± 8.6 nm and 65.5 ± 8.9 nm, respectively, and the zeta potential of ADBMs-P (−42.0 ± 6.4 mV) was significantly less than that of BMs (−28.6 ± 7.6 mV). The optimal encapsulation efficiency and drug loading of Ara-C were 68.4% ± 9.4% and 32.4% ± 2.9%, respectively, and those of DNR were 36.1% ± 2.5% and 17.9% ± 1.6%. Interestingly, this system also exhibits long-term release behaviour sequentially, without an initial burst release. The Ara-C drug continued to release about 85% within 40 days, while DNR release lasted only for 13 days. Moreover, similar to free drugs, ADBMs-Ps are strongly cytotoxic to cancer cells in vitro (HL-60 cells), with the inhibition rate approximately 96%. This study reveals that this new system has a potential for drug delivery application in the future, especially for combination therapy.

## 1. Introduction

Cancer is one of the most common causes of death in humans. Currently, the preferred clinical treatments for cancer include radiotherapy and chemotherapy. Combination drug therapy is administering two or more drugs that provide improved therapeutic efficacy than a single drug, and has become an expedient method of chemotherapy aimed at treating various types of cancers. However, direct drug combination can often exert additional side effects due to their exposure to normal cells. The use of drug carriers can address this issue, as these compounds can eliminate the side effects associated with it and also delivers drugs in a sustained fashion.

Several drug carriers, including liposomes [1], polymer nanoparticles [2] and lipid nanoparticles [3] have been tested in combination therapy. Additionally, researchers are fascinated to the targeted drug carriers because of the simple and controlled targeting mode. Among them, magnetic targeted drug carriers prepared by Fe_3_O_4_ or Fe_2_O_3_ as crystals and biocompatible polymers as envelopes, have attracted considerable interest in the field of drug delivery [4,5,6]. Compared to the synthetic magnetic Fe_3_O_4_ nanoparticles, naturally occurring nanoparticles such as bacterial magnetosomes (BMs) possess greater advantages for biomedical applications. Bacterial magnetosomes are synthesized by magnetotactic bacteria possessing a magnetic iron oxide or iron sulphide core surrounded by a natural organic phospholipid bilayer membrane [7,8,9]. The size of natural BMs is usually between 35 and 100 nm [10]. BMs also possess unique features, such as high chemical purity, narrow size distribution, uniform morphology, and lower toxicity, which make them more desirable for medical use than other types of carriers produced by chemical synthesis [11,12]. In addition, the membrane of BMs possesses active groups and proteins that act as binding sites, while the synthetic magnetic nanoparticles utilize the surface specific active groups either by physical adsorption or chemical combination.

Given the unique features of BMs, particularly the presence of a natural phospholipid membrane, researchers have shown interest in coupling BMs with active substances such as chemotherapeutic drugs [13], peptides [14] and DNA [15,16]. We have reported that BMs can be successfully coupled to cytosine arabinoside (Ara-C) through the surface modification of BMs by poly-l-glutamic acid (PLGA) [17]. We hypothesized that this new system could be used to enhance the antitumor efficacy of drug combination therapy. In this work, Ara-C and daunorubicin (DNR) are used as model drugs to target acute myelogenous leukaemia (AML) illness, to analyse the usefulness of BMs in combination therapy. Dual crosslinkers, including genipin (GP) and PLGA, were used to couple these two drugs to BMs. In this study, we investigated the preparation and characterization of Ara-C-DNR-loaded GP-PLGA-modified bacterial magnetosomes (ADBMs-Ps). The drug-loading and in vitro release profiles, as well as the cytotoxicity of these compounds were also examined in an in vitro model of AML.

## 2. Methods and Materials

### 2.1. Materials

*Magnetospirillum magneticum* AMB-1 was purchased from ATCC (Manassas, VA, USA). Ara-C was purchased from Sunray Pharmaceutical Co., Ltd. (Suzhou, China). DNR was kindly supplied by Meilun Biotech Co., Ltd. (Dalian, China). GP was obtained from Zhixin Biotechnology Company (Fuzhou, China). PLGA was provided by Sigma Aldrich (St. Louis, MO, USA). Unless otherwise specified, all additional chemicals were purchased from Sinopharm Chemical Reagent Co., Ltd. (Beijing, China). The human acute promyelocytic leukaemia cell line, HL-60, was obtained from Typical China Academy Culture Preservation Committee Cell Library (Shanghai, China). Cell culture medium was composed of IMDM supplemented with 20% foetal bovine serum. All cells were incubated at 37 °C in humidified air with 5% CO_2_.

### 2.2. Preparation of the ADBMs-Ps

**Scheme 1:**
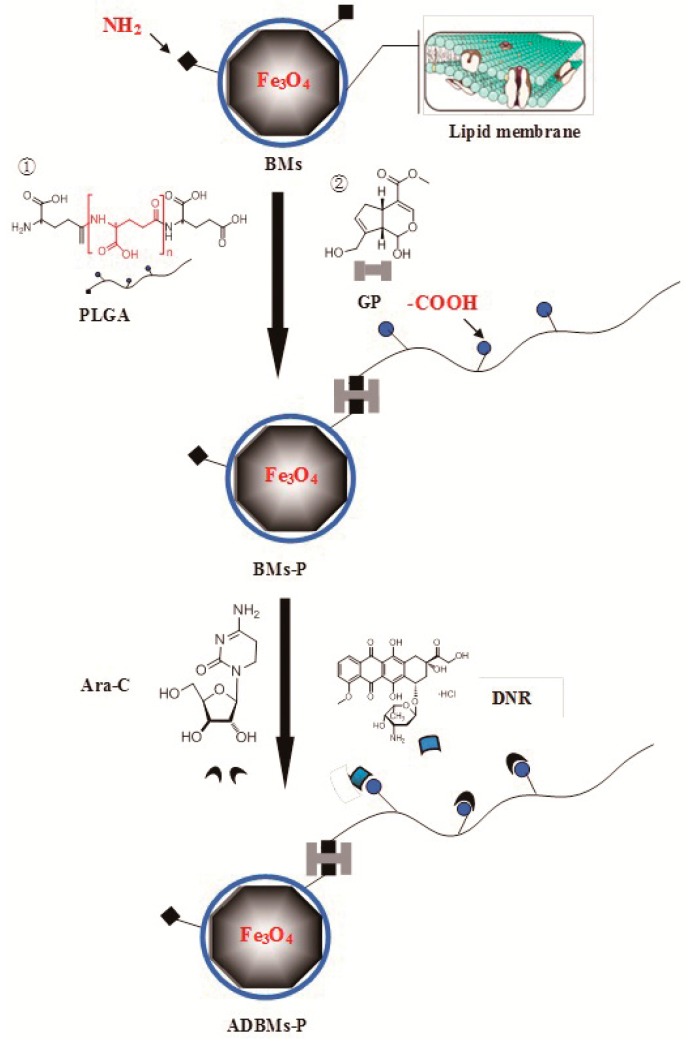
Synthesis of Ara-C-DNR-loaded GP-PLGA-modified bacterial magnetosomes (ADBMs-Ps).

Scheme 1 shows the routes for coupling Ara-C and DNR with BMs by PLGA. The specific steps were as follows. The BMs were derived from AMB-1 cells as previously described [17]. Briefly, AMB-1 cells collected by centrifugation (10,000 rpm for 10 min) were suspended in PBS (pH 7.4) and disrupted by an ultrasonic cell crusher (300 W, worked for 3 s with an interval of 5 s, repeated for 100 times). BMs were collected by a magnet bounded on the bottom of tubes and cell debris was removed. The BMs sediments were collected after being resuspended in PBS (pH 7.4) and being treated with ultrasonic cleaning (40 W, worked for 3 s with an interval of 5 s, repeated for 50 times). This process was repeated 5–10 times. Purified BMs were suspended in PBS (pH 7.4). The same amount of PLGA solution (1 mg/mL) was added to the BMs suspension and the suspension was treated with ultrasonic bathing for 5 min to distribute the BMs evenly. Following the addition of GP solution (the concentration of GP in the mixture was 0.05%, 0.1%, 0.5% or 0.8%), the mixture was distributed by ultrasonic bathing ten times (each lasted for 1 min with an interval of 5 min). The mixture was then placed in an incubator shaker at 60 rpm at 37 °C for 12, 24, 48 or 72 h to react completely. Finally, the PLGA-modified BMs were resuspended in PBS (pH 7.4), with the same addition of Ara-C solution (1 mg/mL), DNR (1 mg/mL) and 1-(3-dimethylaminopropyl)-3-ethylcarbodiimide hydrochloride (EDC) solution (0.2 mg/mL), through sonication (50 W, for 1 min with an interval of 5 min, repeated 10 times). The mixture was then incubated in a shaker at 60 rpm at 37 °C for 12, 24, 48 or 72 h. The ADBMs-Ps were collected and washed to eliminate the residual materials. Finally, the compounds were sterilized before use.

### 2.3. Characterization of the ADBMs-Ps

The ADBMs-Ps were dispersed in distilled water using an ultrasonic bath. The BMs and the previous ADBMs-P suspension (15–20 µL) were loaded on TEM copper grids and examined by TEM. Zeta potential was measured by Zetasizer (ZEN3600; Malvern Instruments Ltd., Malvern, UK). All assays were performed in triplicate. 

### 2.4. The Drug-Loading and Encapsulation Efficiency of ADBMs-Ps

The ADBMs-Ps were distributed in 5 mL of PBS using ultrasonic bathing for 5 min. Then, 200 µL of the ADBMs-P suspensions were taken out and dissolved in 4.8 mL of membrane-breaking liquid composed of 1.2 mL of 36%–38% hydrochloric acid and 3.6 mL of a 70% ethanol solution. The absorption value of the solution was measured at 283.6 and 497.8 nm. The amount of DNR coupled with BMs was represented using a standard curve in membrane-breaking liquid, through the equation *Y* = 32.0855*X* + 0.0963 (*R*^2^ = 0.9999), where *Y* is the concentration of DNR (µg/mL) and *X* is the absorbance of DNR in membrane-breaking liquid at 497.8 nm. The amount of Ara-C was calculated by the double-wave method, with the standard curve equation *Y* = 68.6690*X* + 0.0570 (*R*^2^ = 0.9995), where *Y* is the concentration of the mixture (µg/mL) and *X* is the absorbance of the mixture in membrane-breaking liquid at 283.6 nm.

### 2.5. In Vitro Release Studies

The in vitro release studies were performed by placing the sample filled tubes in a rotary shaker (37 °C, 60 rpm). The drug (Ara-C or DNR) content in the supernatant of each tube was evaluated by UV-vis spectrometer after incubation for 0.5, 1, 12, 24, 36 or 48 h. Equal amounts of PBS (2 mL) were then added into the tubes. The concentration of DNR in the PBS was calculated through the following standard curve: *Y* = 46.1316*X* + 0.1796 (*R*^2^ = 0.9998), where *Y* is the concentration of DNR (µg/mL) and *X* is the absorption of DNR in PBS at 484.4 nm. The amount of Ara-C was calculated by the double-wave method, with the standard curve equation: *Y* = 50.1865*X* + 0.0166 (*R*^2^ = 0.9999), where *Y* is the concentration of the mixture (µg/mL) and *X* is the absorbance of the mixture in PBS at 271.4 nm. All assays were performed in triplicate.

### 2.6. Cytotoxicity Assay

The samples of Ara-C, DNR, BMs, and ADBMs-Ps were sterilized by ^60^Co irradiation (7.5 kGy) before use. The Ara-C content with adequate IMDM medium for control was 0.1, 1, 5, and 25 μg/mL, respectively. To investigate the influence of incubation time, the drug concentration was 1 μg/mL. BMs were used as a control and adjusted to the same concentration as others. The CCK-8 assays were performed to study the effects of drug concentration and incubation time on cytotoxicity according to the manufacturer’s instructions.

## 3. Results and Discussion

### 3.1. Characterization of the ADBMs-Ps

**Figure 1:**
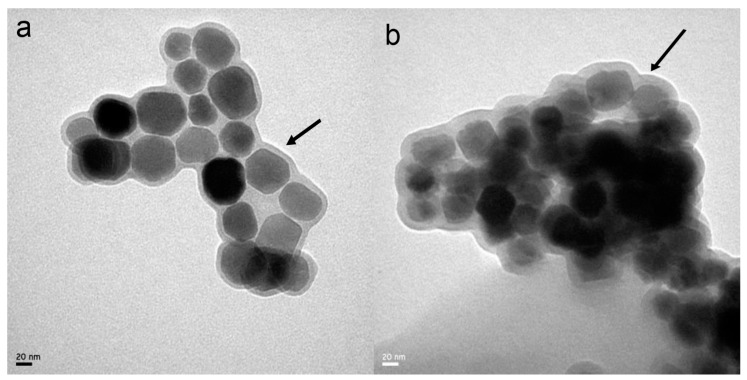
TEM images of: bacterial magnetosomes (BMs, **a**); and ADBMs-P (**b**). Black arrows showL uniform and clear lipid membranes of BMs (**a**); and thickened and blurred membranes of ADBMs-P (**b**).

In general, the crosslinking agents such as glutaraldehyde increase the potential toxicity of the future applications. Herein, we developed a drug delivery system by the utilization of a natural dual functional crosslinker GP, which has the advantages of biocompatibility, low toxicity, and some pharmaceutical properties and will broaden the application of BMs. As illustrated in Figure 1, both BMs and ADBMs-Ps were surrounded by lipid membranes. In addition, the size distribution and morphology of ADBMs-Ps were uniform and consistent with that of BMs. However, a thick, nebulous material, which was presumed to be the Ara-C and DNR coupling, appeared on the outside of ADBMs-Ps. The characterization by TEM showed that the diameter of ADBMs-P was larger than that of BMs (see Table 1). Consistent with previous studies, [18,19,20] these findings suggest that Ara-C and DNR might attach to the lipid membrane of BMs. We also found in the experiment that the suspend solution was stable and transparent, which might be due to the electrostatic repulsion of the lipid membranes [21]. This phenomenon is consistent with the zeta potential values (Table 1), which suggest that the natural nanoparticles are stable in the solution.

### 3.2. The Drug-Loading and Encapsulation Efficiency of ADBMs-Ps

**Figure 2:**
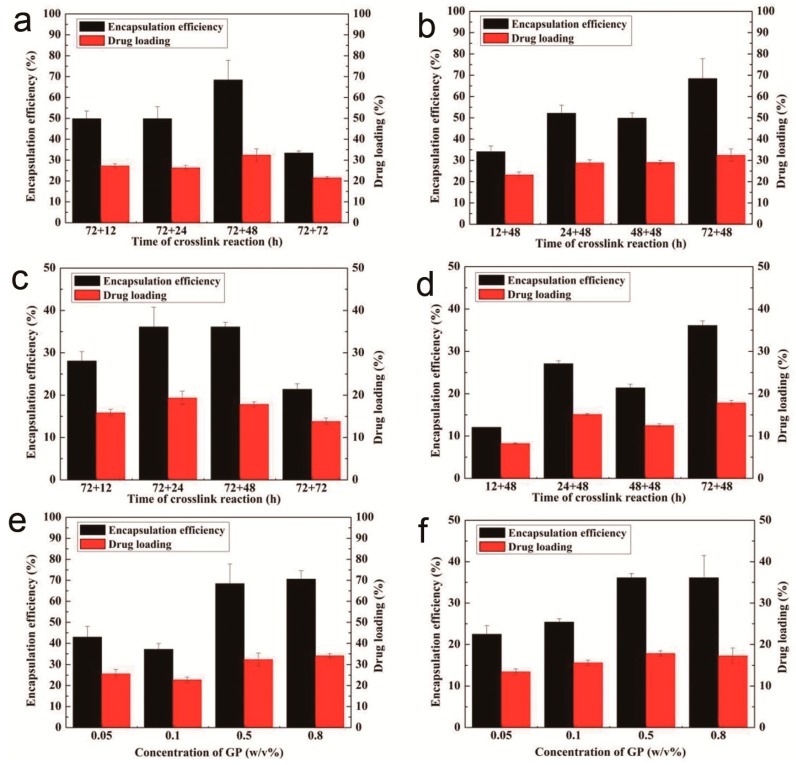
Drug-loading and encapsulation efficiency of ADBMs-P preparation with different crosslinking reaction times: (**a**,**b**) Ara-C; and (**c**,**d**) DNR; and different GP concentration: (**e**) Ara-C; and (**f**) DNR.

It is well accepted that the drug-loading properties of the carriers can influence the therapeutic efficacy. As evidenced in Figure 2, crosslinking reaction times greatly influenced the drug-loading and encapsulation efficiency of ADBMs-Ps. Figure 2a,c shows that both the drug-loading and encapsulation efficiency attained an optimal value (32.4% ± 2.9% and 68.4% ± 9.4%, respectively for Ara-C and 17.9% ± 1.6% and 36.1% ± 2.5%, respectively for DNR) when the first-step crosslinking reaction lasted for 72 h and the second for 48 h. A tendency for lower drug-loading and encapsulation efficiency was obtained when the second-step crosslinking reaction time was longer because the drugs were coupled to BMs through the chemical crosslinking reaction and physical absorption, and some drugs released during the second-step crosslinking reaction because of surface absorption. Compared with our previous study [17], a small decrease in drug-loading of Ara-C was found due to the competitive occupying of the efficient combined sites by DNR. Nevertheless, the drug-loading and encapsulation efficiency of DNR was lower than Ara-C because of much longer coupling evaluation, i.e., a maximum drug-loading obtained within several hours [22]. Another reason might be the more complex loading procedure of DNR through EDC-coupled onto the BMs’ surface.

As Figure 2b,d shows, both the drug-loading and encapsulation efficiency reached a maximum value when the second-step crosslinking reaction time was 48 h and the first-step crosslinking reaction time was 72 h. However, when the first-step crosslinking reaction lasted for 48 h, both the drug-loading and encapsulation efficiency showed a decreasing trend which were lower than the conditions stated above. In addition, PLGA may form a gel-like substance that surrounds BMs, which further inhibits the coupling of ADBMs-Ps with the drug. In Figure 2e,f, an enhancement of the drug-loading and encapsulation efficiency can be seen with the increasing GP concentration. When the concentration reached 0.5%, the drug-loading process reached equilibrium. Interestingly, the total drug-loading of ADBMs-Ps reached 51.6%, which is higher than the reported [18,19,23].

From the above results, it is clear that the amount of drug loaded on BMs varies with crosslinking times and the concentration of crosslinkers. The total drug-loading is much higher than that of synthetic magnetite which may be due to the abundant active sites on the outer membrane of BMs.

### 3.3. In Vitro Release Studies

**Figure 3:**
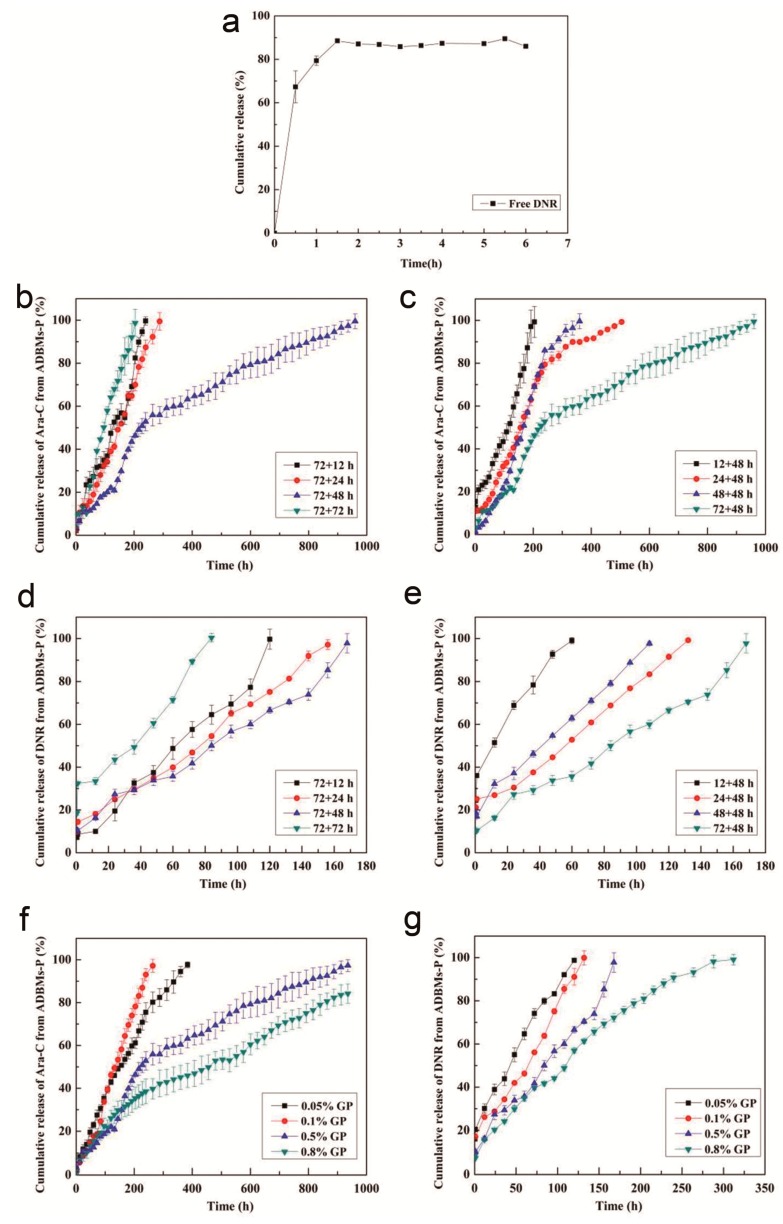
Cumulative release of: (**a**) DNR; and ADBMs-P preparation with different reaction time: (**b**,**c**) Ara-C; and (**d**,**e**) DNR; and different GP concentration: (**f**) Ara-C; and (**g**) DNR.

As shown in Figure 3, the solubility of free drug was fast (Figure 3a), whereas the release from ADBMs-P was significantly slower. Figure 3b–e shows that, when the first-step crosslinking reaction lasted for 72 h, and the second crosslinking step lasted for 48 h, the slowest release behaviour was observed. Ara-C continued to be released at about 85% within 40 days while DNR released lasted only 13 days. This is because a thorough crosslinking reaction occurred when the crosslinking reaction times were 72 and 48 h; therefore, at these time-points, Ara-C and DNR were closely linked to BMs, producing a longer release period. While when the reaction time was 72 + 72 h, the cumulative release got faster because some drugs have been released in the later stage of the reaction. As demonstrated in Figure 3f,g, the GP concentration also affected the release property, as slower release behaviour was observed with increasing concentrations of GP. This is presumed to occur because the drug content coupled with BMs is higher with the increasing of GP concentration, which confers a longer release property, while previous in vitro release studies mainly focused on short-term release behaviour [20,22,24]. Therefore, we successfully constructed a magnetic targeting antitumor drug delivery system with long-term release behaviour with no burst release effect.

### 3.4. Cytotoxicity Assay

**Figure 4:**
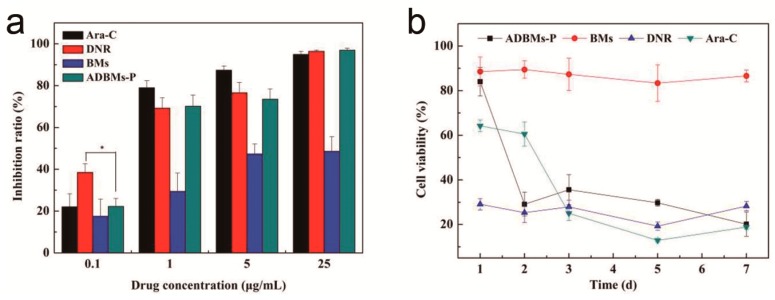
The effect of: concentrations (**a**); and treatment times (**b**) of Ara-C, DNR, ADBMs-P and BMs on the growth of HL-60 cells (* *p* < 0.05).

The antitumor effect of ADBMs-Ps was evaluated using HL-60 cells, a model of AML. The inhibition rates of Ara-C, DNR, ADBMs-P, and BMs showed a rising trend with increasing drug concentrations (Figure 4a). When the drug concentration reached 25 µg/mL, the ADBMs-Ps inhibited cell proliferation. The effect of ADBMs-Ps on the cell growth inhibition rate increased to 96%, which was higher than that of the control group (Ara-C and DNR alone) and these reports are in agreement with the previous reports [19]. 

Experiments examining various incubation times indicated that the BMs group maintained a high level of cell viability throughout the incubation period (Figure 4b). At the same time, the Ara-C, DNR, and ADBMs-P groups showed obvious cytotoxicity compared with the BMs group. In the Ara-C group, cell growth was inhibited and viability continued to decline for five days followed an upward trend later. The reason might be the initial concentration of Ara-C was sufficient to inhibit cell proliferation significantly but failed to kill the cells completely. After five days, the surviving cells began a new round of proliferation. The inhibition effect of DNR was stronger, and it showed the same change trend as the Ara-C group. The inhibition rate of ADBMs-Ps was strengthened following prolonged incubation. The activity of cells decreased to 20% on Day 7 and was very near those of the un-conjugated Ara-C and DNR drugs. This is due to the long-term release property of ADBMs-Ps. Therefore, we predict that the ADBMs-Ps would continue to suppress cell proliferation given longer incubation times. Furthermore, other researchers have reported that BMs are biocompatible both in vitro and in vivo and suggested that the magnetosomes also have the potential as novel drug or gene carriers for tumour therapy [25,26].

## 4. Conclusions

In summary, the strategy to construct an ADBMs-P magnetic targeted delivery system was efficient and successful, which led to preferable drug-loading and encapsulation efficiency (Ara-C and DNR) within BMs membranes using dual crosslinkers. ADBMs-Ps exhibited long-term release ability and stability, while no initial burst release behaviour was exhibited. Moreover, the ADBMs-P was strongly cytotoxic to HL-60 cells, which could make this an appropriately controlled drug release system and yielded an improved therapeutic effect along with minimized side effects of the drugs. The results also demonstrated that GP and PLGA could be used to couple two kinds of drugs with BMs membranes. However, the role of the BMs membrane has not been fully explored. The results presented here demonstrated a positive step towards expanding the scope of applications of BMs within the medical field.

## Figures and Tables

**Table 1:** Size and zeta potential of BMs and ADBMs-P.

Type	Size (nm)	Zeta Potential (mV)
BMs	42.0 ± 8.6	–28.8 ± 7.6
ADBMs-P	65.5 ± 8.9	–42.0 ± 6.4
